# Standardization to Characterize the Complexity of Vessel Network Using the Aortic Ring Model

**DOI:** 10.3390/ijms26010291

**Published:** 2024-12-31

**Authors:** Petra Wolint, Silvan Hofmann, Julia von Atzigen, Roland Böni, Iris Miescher, Pietro Giovanoli, Maurizio Calcagni, Maximilian Y. Emmert, Johanna Buschmann

**Affiliations:** 1 Division of Surgical Research, University Hospital of Zurich, 8091 Zurich, Switzerland; 2 Department of Plastic Surgery and Hand Surgery, University Hospital Zurich, 8091 Zurich, Switzerland; silvan.hofmann@uzh.ch (S.H.); julia.vonatzigen@usz.ch (J.v.A.); iris.miescher@usz.ch (I.M.); pietro.giovanoli@usz.ch (P.G.); maurizio.calcagni@usz.ch (M.C.); 3 White House Center for Liposuction, 8044 Zurich, Switzerland; info@whitehousecenter.ch; 4 Institute for Regenerative Medicine (IREM), University of Zurich, 8952 Zurich, Switzerland; emmert@dhzb.de; 5 Deutsches Herzzentrum der Charité (DHZC), Department of Cardiothoracic and Vascular Surgery, 13353 Berlin, Germany; 6 Charité-Universitätsmedizin Berlin, Corporate Member of Freie Universität Berlin and Humboldt-Universität zu Berlin, 13353 Berlin, Germany; 7 BIH Center for Regenerative Therapies (BCRT), Berlin Institute of Health at Charité-Universitätsmedizin Berlin, 13353 Berlin, Germany

**Keywords:** angiogenesis, aortic ring assay, secretome, mesenchymal stem cells, spheroid, index

## Abstract

Regeneration after ischemia requires to be promoted by (re)perfusion of the affected tissue, and, to date, there is no therapy that covers all needs. In treatment with mesenchymal stem cells (MSC), the secretome acts via paracrine mechanisms and has a positive influence on vascular regeneration via proangiogenic factors. A lack of standardization and the high complexity of vascular structures make it difficult to compare angiogenic readouts from different studies. This emphasizes the need for improved approaches and the introduction of an index in the preclinical setting. A characterization of human MSC secretomes obtained from one of the three formats—single cells, small, and large spheroids—was performed using the chicken aortic ring assay in combination with a modified angiogenic activity index (AAI) and an angiogenic profile. While the secretome of the small spheroid group showed an inhibitory effect on angiogenesis, the large spheroid group impressed with a fully pro-angiogenic response, and a higher AAI compared to the single cell group, underlying the suitability of these three-stem cell-derived secretomes with their distinct angiogenic properties to validate the AAI and the novel angiogenic profile established here.

## 1. Introduction

Angiogenesis is a fundamental process for diverse physiological and pathological mechanisms in tissue regeneration [1,2]. New blood vessels are formed from the existing vasculature [3] and thereby maintain blood flow to supply cells with oxygen and nutrients [4,5,6]. After ischemia, the body attempts to regenerate the tissue through perfusion [4]. Appropriate intervention due to pharmaceuticals or therapeutic stem cell products supports the formation of new blood vessels [5]. This is of great importance in a variety of diseases that affect millions of people worldwide [5,7].

Cell-based therapeutic approach involves secreted factors of mesenchymal stem cells (MSCs), which is an extensively investigated cell type candidate for vascular regeneration, acting through a paracrine mechanism [8,9]. Several pro-angiogenic factors of MSCs have been reported in the literature to induce angiogenesis, including vascular endothelial growth factor (VEGF) [8,9,10], hepatocyte growth factor (HGF) [8,11,12], insulin-like growth factor-1 (IGF-1) [8], fibroblast growth factor (FGF) [9], transforming growth factor-alpha (TGF-α) [11,13], transforming growth factor-beta (TGF-β) [11,14], and tumor necrosis factor-alpha (TNF-α) [11,15]. By stimulating the revascularization in this way, endothelial cells (ECs) in the body can sprout from a root vessel, migrate and proliferate [16,17,18]. The process continues with the alignment of ECs and ends with the formation of new vessels [16,17,18]. Furthermore, by aggregating stem cells, for example, MSCs, into multicellular three-dimensional (3D) spheroids in vitro, the resulting cell–cell interactions are enhanced compared to a monolayer culture [19,20]. Such microtissues have a hypoxic core and the hypoxia-driven secretion has been reported to lead to higher amounts of e.g., VEGF and FGF [21,22], thus increasing angiogenic efficacy [21,23].

Today, cell-free approaches are becoming increasingly important in the field of regenerative medicine due to the lack of engraftment and proliferation of the applied MSCs [24,25,26,27,28,29]. Bioactive molecules, soluble proteins, free nucleic acids, lipids, and extracellular vesicles released by the cells to the extracellular space constitute the secretome [30,31,32,33,34]. The advantages as a therapeutic product are lower immunogenicity, easier storage, and simpler handling compared to the cell-based approach [30,31,32,33,34]. A variety of pre-clinical studies to date, demonstrated the efficacy of transplanted secretome [34]. Timmers and his co-workers have shown a reduction in myocardial infarct size in a porcine and mouse model of ischemia and reperfusion injury [35]. In clinical trials, bone marrow-derived MSCs, among others, have already been used to improve alveolar bone regeneration [34,36].

To investigate and assess the angiogenic potential of secretome, numerous bioassays have been developed [37]. In vitro assays for angiogenesis cannot reflect the entire angiogenic process [37]. To further create a more in vivo situation these in vitro assays have been combined with organ culture techniques. One example of this approach is the aortic ring assay. In contrast to the in vitro assays working only with isolated endothelial cells, the ex vivo aortic ring model incorporates other cell types like pericytes, macrophages, and fibroblasts [38]. Furthermore, endothelial cells of an aortic ring explant do not need to be isolated, causing a potential change in their properties [38]. The assay allows the evaluation of many different parameters, enabling the observation of several aspects of an experimental setup and its outcome. To perform an aortic ring assay, a suitable matrix for the ring placement must be chosen. One prominent representative is matrigel, which was developed from Engelbreth–Holm–Swarm (EHS) mouse sarcoma cells more than 30 years ago and it is used extensively for angiogenic bioassays up to date [39]. Although it is commonly utilized in the study of cell differentiation, tumor growth, and angiogenesis, some features of matrigel are disadvantageous. Matrigel has an ill-defined and variable composition [40]. The interest in matrigel alternatives has increased over the last years, resulting in the production of matrigel-free solutions [41]. Mousseau et al. described egg white as a new alternative to matrigel used as a matrix for angiogenesis assays [42]. Another matrix that was developed in recent years is jellagel. This collagen hydrogel is manufactured from jellyfish. Therefore, unlike matrigel, jellagel lacks ethical concerns and by its more defined composition promises more consistent and reproducible results [43].

Various evaluation methods and parameters are available to assess angiogenic potential [11,37,38]. It is possible to perform the analysis of microscopic images manually or automated by means of specific programs [16,44,45,46]. Diversity is a challenge [46] firstly in terms of different terminologies based on a common definition, and secondly standardizable [47] in terms of quantitative assessment. Table A1 provides an overview of the current literature, and the various terms used. The different terminologies, which can be assay-specific or assay-overlapping, are a pitfall for the direct comparability of preclinical results. In the field of cancer diagnostics, an index has already been developed by Demir and co-workers for the standardized evaluation of antiangiogenic drugs in the preclinical phase [47]. Using the example of bevacizumab, which has regulatory approval for the therapy of metastatic colorectal carcinoma, the drug angiogenic activity index (DAAI) was presented by means of the chorioallantoic membrane (CAM) assay [47]. The DAAI allows comparability and assessment of drug screenings and enables translation of the impression of potential desirable and adverse clinical effects of antiangiogenic agents [47]. Other known angiogenic indices refer to isolated specific foci such as microvessel count (vessels per square millimeters or per field) [48,49], immunohistochemical cluster differentiation (CD) 31-positive vessel staining [50], or vascular intersections on a grid [51] and are neither cross-method nor to be understood as a comprehensive assessment of angiogenic activity. In regenerative medicine, there are also indices, but they are not explicitly tuned to angiogenesis alone and are considered in the overall clinical picture. For this purpose, the outcome score named integrated multimodal potency analysis of cardiac cell therapy (IMPACCT) was developed, which is of essential value in the quantitative evaluation of myocardial regeneration in regard to assessing the safety and efficacy of a cell-based therapy [52]. The IMPACCT comprises a group of 24 parameters that include both functional and pathological references, as well as determined biomarkers [52]. Although different indices, from very simple [47,48,49,50,51] to complex [52], already exist, they are hardly used. In contrast to the large number of preclinical studies performed, not enough conclusions can be drawn from them. Mostly, very specific expertise is required to relate and compare different in vitro-based angiogenesis studies to each other. All the more, there is a need for a common language, an index that harmonizes the assessment of outcomes across studies and makes them accessible to a broad audience.

In the present study, we investigated (1) different matrices for the angiogenic potential assessment using the chicken aortic ring assay, (2) the angiogenic potential of human MSC-derived secretomes from the following three different cell formats: single cell (SCs) monolayers, small and large 3D-spheroid cultures, (3) the influence of spheroid size on vascularization, and finally introduced a modified angiogenic activity index (AAI) combined with a novel individual angiogenic profile to ensure standardization of results of the analyzed complex microvascular network.

## 2. Results

### 2.1. Evaluation of Different Matrices for Chicken Aortic Ring Assay

**Figure 1:**
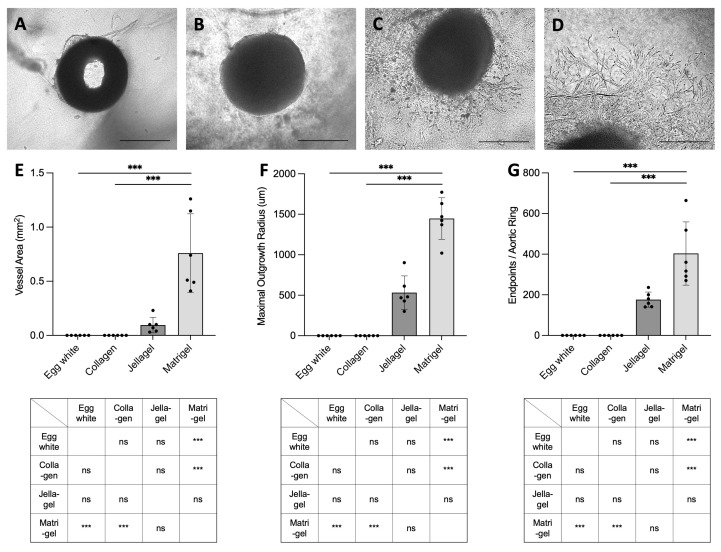
Comparison of different matrices used for aortic ring assay. The following four different matrices were compared using a complete medium: chicken egg white (**A**), collagen from bovine skin (**B**), jellagel (**C**), and matrigel (**D**). The parameters shown in bar charts are the calculated vessel area (**E**), maximal outgrowth radius (**F**), and endpoints per aortic ring (**G**). Scale bars 500 μm (**A**–**D**). Data shown are means ± SD with individual values (*n* = 6). Groups were compared using nonparametric Kruskal–Wallis test (ns: not significant; *** *p* < 0.001).

In order to select the most suitable matrix for evaluation of the secretome using the chicken aortic ring assay, the four matrices described in the literature were compared (Figure 1). After seven days no outgrowth of vascular sprouting from the aortic ring was observed with either the chicken egg white (Figure 1A) or collagen matrix (Figure 1B) when cell culture medium containing FCS was used. The vessel area (Figure 1E) was not equally pronounced in the jellagel matrix as in the matrigel matrix (0.0950 ± 0.0723 vs. 0.760 ± 0.363 mm^2^). The same tendency in favor of matrigel was seen for the maximal outgrowth radius (532 ± 207 μm for jellagel vs. 1448 ± 257 μm for matrigel; Figure 1F) as well as for the number of endpoints per aortic ring (176 ± 37 for jellagel vs. 404 ± 156 for matrigel; Figure 1G). Due to the statistically significant results of matrigel versus the egg white and collagen matrices (*p* < 0.001; Figure 1E–G), only matrigel was chosen for the subsequent experiments.

### 2.2. Parameters for the Assessment of Explants and Determination of Sprouting Pattern

**Figure 2:**
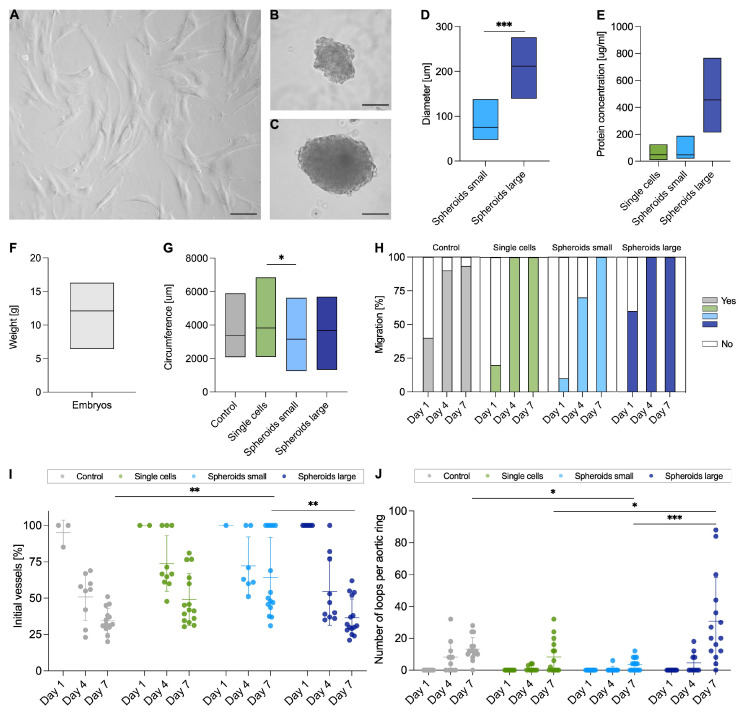
Collecting of secretomes, comparability of chicken aortas, and pattern analysis. For the production of the secretomes, human adipose-derived mesenchymal stem cells were cultivated either as single cells ((**A**); scale bar 100 μm) or as spheroids small ((**B**); scale bar 50 μm) and spheroids large ((**C**); scale bar 50 μm). After 3 days, the diameter of the spheroids was determined (**D**). The protein concentration of the harvested secretomes was measured (**E**). Before harvesting the aorta, each chicken embryo was weighed (**F**) and for the aortic rings used in the aortic ring assay, the circumference was measured under the microscope (**G**). Migration of the aortic rings was determined on days 1, 4, and 7, and visible vessel sprouting was considered positive (Yes; (**H**)). Vessel architecture was assessed by primary initial vessels in relation to total vessel number (**I**) and by the number of loops formed (**J**). The aortic rings were treated with either serum-free medium (Control), secretomes of single cells, spheroids small or spheroids large (**G**–**J**). Data are shown as boxplots with min to max values and mean (**D**–**G**). Groups were compared using Mann–Whitney test (**D**), one-way ANOVA with Tukey’s multiple comparison tests (**G**), and nonparametric Kruskal–Wallis test for day 7 time point (**I**,**J**); * *p* < 0.05; ** *p* < 0.01; *** *p* < 0.001.

For the production of secretomes, human adipose-derived MSCs were either grown as single cells (Figure 2A) or cultivated as small (Figure 2B) or large spheroids (Figure 2C) using the hanging drop method. After three days, the spheroids small had a diameter of 78 ± 26 μm compared to the spheroids large with a diameter of 212 ± 42 μm (*p* < 0.001; Figure 2D). After incubating the SCs or 3D spheroids in a serum-free medium, the secretomes of the three groups of SCs, spheroids small and spheroids large were harvested. The total protein concentration was determined for each secretome group (E).

Before harvesting the aorta, each chicken embryo was weighed (12.1 ± 2.8 g; Figure 2F). By determining the weight, it was ensured that all embryos were at a comparable stage of development. The size of aortic explants embedded in the matrix was determined by the circumference under the microscope (Figure 2G). A significantly larger diameter (*p* < 0.05) was determined for the single cells group (3821 ± 1048 μm) compared to the spheroids small group (3156 ± 925 μm). No significant difference could be determined for the other groups (control group: 3386 ± 912 μm, spheroids large group: 3671 ± 1153 μm). Vessel sprouting was observed from day 1 onwards in all groups of individual explants, which was detected in all explants used up to day 7 except for the control group (Figure 2H). In the single cells and spheroids large groups, migration was observed in all explants used from incubation day 4 onwards.

Pattern analysis involves determining the ratio of primary vessels formed by sprouting from the explant to the total number of vessels present (Figure 2I). As seen in the example of the spheroids large group, only primary vessels were detected after one day of incubation (100 ± 0%). On day 4, the percentage of primary vessels was 54.8 ± 23.2%, and by day 7, this value decreased further (36.5 ± 12.9%) due to further growth of branches. Comparison of day 7 time points showed significance for the control and spheroid small groups (*p* < 0.01, 34.4 ± 8.5% and 64.2 ± 27.5%), and spheroid small and spheroid large (*p* < 0.01) groups. Loop formation (Figure 2J) was most pronounced at day 7 for the spheroids large group with 30.7 ± 27.4 loops per ring. This was significant over the single cells group with *p* < 0.05 and over the spheroids small group with *p* < 0.001. A significance could also be determined between the control and spheroid small groups (*p* < 0.05) in favor of the spheroid small group.

For all four groups, a dependence over time, measured at 1, 4, and 7 days after the beginning of incubation, was detected for both initial vessel formation and loop structure.

### 2.3. Analysis of the Network Characteristics Originating from the Aortic Explant

**Figure 3:**
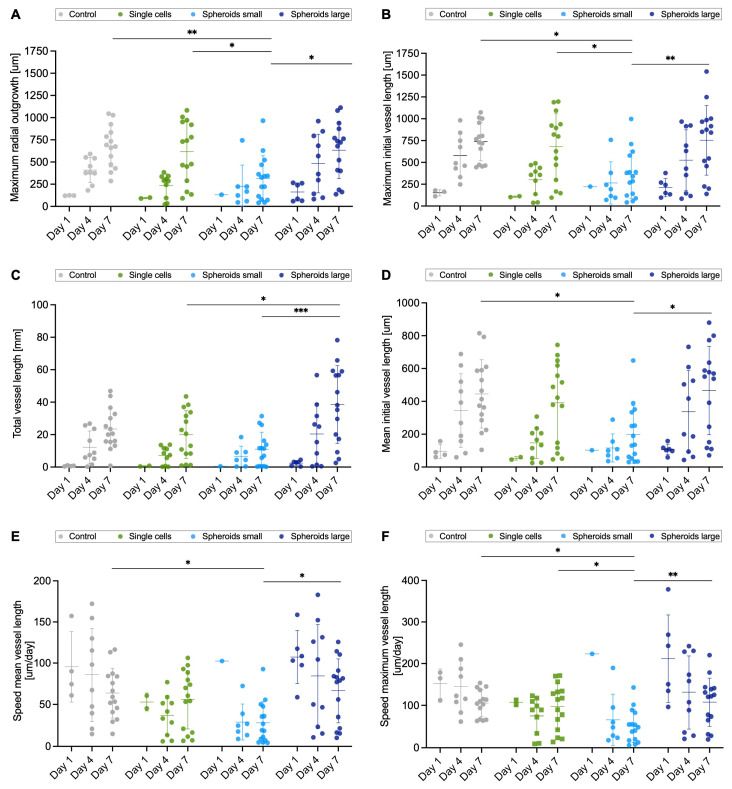
Comprehensive network characterization. The parameters maximum radial outgrowth (**A**), maximum initial vessel length (**B**), total vessel length (**C**), mean initial vessel length (**D**), speed mean initial vessel length (**E**), and speed maximum initial vessel length (**F**) were determined for the groups (**A**–**F**) control, single-cells secretome, spheroids small secretome, and spheroids large secretome at time points day 1, 4 and 7, respectively. Groups were compared using one-way ANOVA with Tukey′s multiple comparison tests (**A**–**C**,**F**) and nonparametric Kruskal–Wallis test for day 7 time point ((**D**,**E**); * *p* < 0.05; ** *p* < 0.01; *** *p* < 0.001).

The maximum radial outgrowth (Figure 3A) showed the highest values (680 ± 235 μm for the control group, 617 ± 343 μm for single-cells group, 317 ± 256 μm for spheroids small group, 633 ± 310 μm for spheroid large group) in each group on day 7. In contrast to the radius measured at an angle of 90 degrees to the aortic ring, the maximum initial vessel length (Figure 3B) showed slightly higher values due to the vessel not growing straight. The following values were determined on day 7 for the control group, the single-cells group, the spheroids small group, and the spheroids large group: 739 ± 221 μm, 680 ± 378 μm, 361 ± 272 μm, and 754 ± 400 μm.

For the parameter of total vessel length, which includes the sum of all vessel lengths together with the branches, the spheroids large secretome group shows significant differences from the single-cells secretome (*p* < 0.05) and spheroids small secretome (*p* < 0.001) groups on day 7 (Figure 3C). When analyzing the mean of all initial vessels, the mean vessel length changes toward day 7 with respect to an increasing size (Figure 3D). When calculating the speed of both the average initial vessel length (Figure 3E) and the maximum initial vessel length (Figure 3F), it was observed that the longer the incubation period, the slower the growth of micrometers per day. Except for the single-cell group (Figure 3E,F), a change in velocity over time was observed in all other groups.

### 2.4. The Evaluation of Branches and Junctions for Determination of Sprouting Parameters

**Figure 4:**
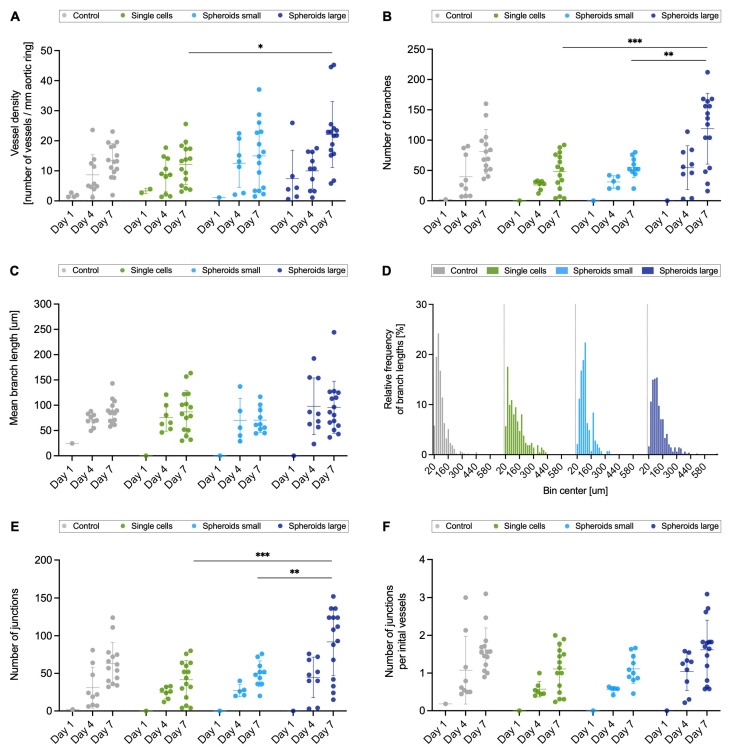
Comparison of sprouting parameters. The sprouting was compared between the groups: control, single cells, spheroids small, and spheroids large, and for this purpose the parameters vessel density (**A**), number of branches (**B**), mean branch length (**C**), relative frequency of branch lengths (**D**), number of junctions (**E**), and number of junctions per initial vessels (**F**) were analyzed. Groups were compared using one-way ANOVA with Tukey’s multiple comparison tests (**B**,**E**,**F**) and the nonparametric Kruskal–Wallis test for the day 7 time point ((**A**,**C**); * *p* < 0.05; ** *p* < 0.01; *** *p* < 0.001).

With the initial vessels counted vessel density per millimeter of aortic ring circumference was calculated (Figure 4A). An increase in vessel density was recorded over the 7 days with significance between the single-cell and spheroids large groups (*p* < 0.05). The determined branches were analyzed in depth with respect to number (Figure 4B), average length (Figure 4C), and size distribution (Figure 4D). The number of branches on day 7 showed the following values for the groups: control, single cells, spheroid small, and spheroid large: 81.1 ± 36.4, 48.1 ± 30.6, 55.2 ± 17.3, and 119 ± 58.4. No significant differences were found in the average branch length. This is supported by the qualitative representation of the size frequencies (Figure 4D). The same pattern is seen in all four groups, although, it is noticeable that the single cells and spheroid large groups have isolated longer branches. The majority of all branches are around 100 μm in length for all groups on day 7. The number of junctions showed significance between the spheroid large group and the single-cell (*p* < 0.001) and spheroids small (*p* < 0.01) groups. If the number of junctions is normalized to the initial vessels, the following values per initial vessels are recorded for day 7: 1.61 ± 0.584 for the control group, 1.11 ± 0.602 for the single-cell group, 1.11 ± 0.388 for the spheroid small group, and 1.62 ± 0.781 for the spheroid large group. Sprouting is seen in all groups from the fourth day of incubation and shows a similar trend between groups regardless of the parameter determined.

### 2.5. Quantification of Angiogenesis Based on Angiogenic Activity Index (AAI)

**Figure 5:**
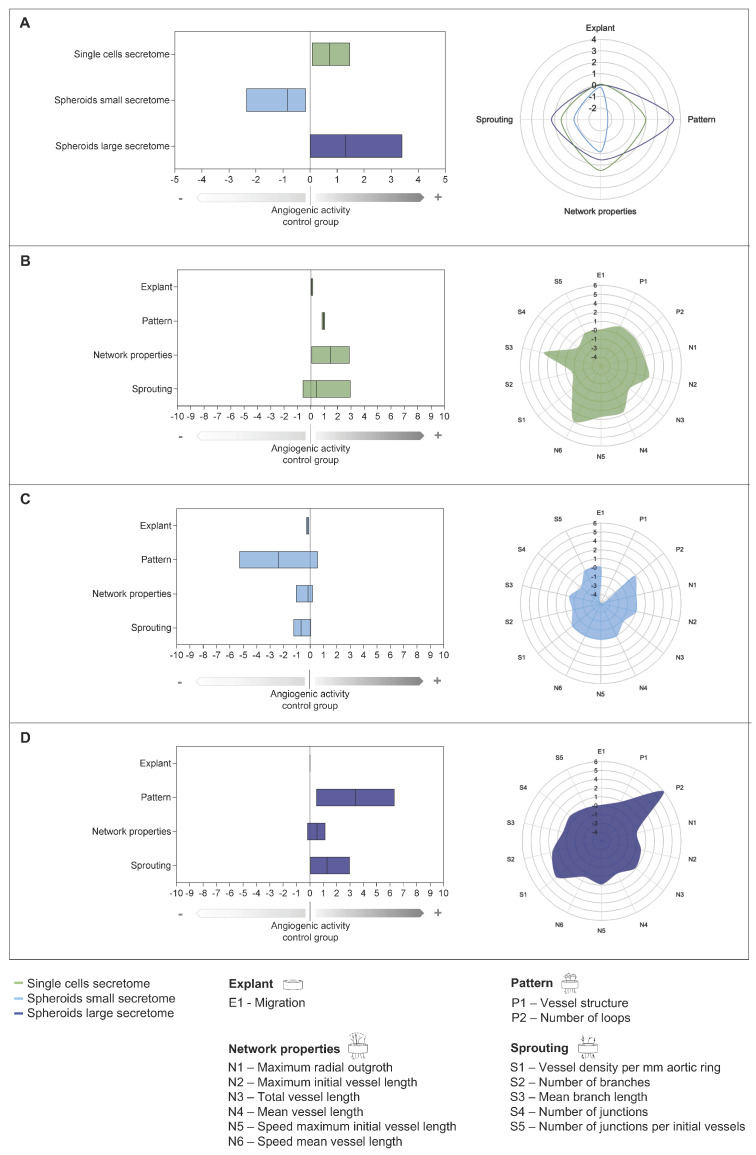
Comprehensive characterization of secretome groups using angiogenic activity index (AAI) and angiogenic profile. The AAI for the secretome groups single cells, spheroid small and spheroid large (**A**), as well as for each group individually single cells (**B**); spheroid small (**C**); spheroid large (**D**) with the presentation of the subindex categories. Data are shown as boxplots with min to max values and mean. The value zero represents the angiogenic activity of the control group (serum-free medium) and values in the positive scale range indicate an increase in angiogenic activity compared with the control group, with a value of 1 representing an increase of 100%. The scale range with negative values shows a decrease or inhibition of angiogenic activity. The angiogenic profile is plotted in the chart and splits the values from the boxplot into the subindex categories (**A**). The individual parameters of the three examined secretome groups: single cells ((**B**), shown in green), spheroids small ((**C**), shown in light blue), and spheroids large ((**D**), shown in dark blue) are shown in the respective radar chart.

For the calculation of AAI and the establishment of an angiogenic profile, the values of days 4 and 7 were included in the analysis. For this purpose, the measured parameters (Figure 2, Figure 3 and Figure 4) were first determined for each donor and each corresponding secretome group and negative control. The calculation was then performed according to Equation (1), and the mean value was calculated from each secretome group for the respective parameter investigated (Table S1). The parameters combined with the subindex were calculated again as a mean value. Isolated parameters denoted by a predictive importance factor of two were weighted higher for the assessment of angiogenic activity, and this was also included in the calculation of the final AAI (Table S1). This step-by-step procedure allows, first, a statement about the resulting network (Figure 5B–D) based on the four subindex categories explant, pattern, network properties, and sprouting and, second, the calculation of the final AAI, which reflects the overall angiogenic activity (Figure 5A), taking into account the aforementioned categories. According to the calculations, the value zero in the AAI corresponds to the values of the control group, while the value 1 corresponds to an increase (for a value of +1) and a diminution (for a value of −1), respectively, in the measured angiogenesis. The final AAI was calculated to be 0.732 for the single cells secretome group, −0.841 for the spheroids small secretome group, and 1.312 for the spheroids large secretome group (Table S1).

The graphical representation of the results from Table S1 is summarized in Figure 5. The spheroids small group showed a decrease in angiogenesis compared to the control group, but also compared to the other two secretome groups, single cells and spheroids large (Figure 5A,C). Already during migration, only this group showed explants, which did not sprout even after 7 days. The two groups, single cells and spheroids large, both showed pro-angiogenic values with a corresponding profile (Figure 5A–D). The main focus of the single cells was sprouting, whereas the subindex pattern was more pronounced in the spheroid large group. In the single-cell group, parameters with an anti-angiogenic tendency were also included in the AAI, which resulted in a final score lower than that of the two spheroid secretome groups, despite a noticeable sprouting. The values for parameters of the spheroid large group were exclusively in the proangiogenic range and thus resulted in a completely pro-angiogenic profile covering all areas of the subindices, although the network properties with maximum radial outgrowth and mean vessel length did not reflect strengths of the angiogenic profile (Figure 5D).

In addition to the standardized measurability of angiogenic activity with the AAI, the angiogenic profiles for each of the investigated secretome groups single cells, spheroids small, and spheroids large show the individual characteristic angiogenic properties. Thereby, the group spheroids large was identified to have the most complete proangiogenic efficacy (Figure 5A,D).

## 3. Discussion

In regenerative medicine, MSCs have been used as a cell-based therapy for some time, and their efficacy has been extensively studied [53,54,55], including their ability to regenerate vascular tissue [56,57,58,59,60,61,62]. Secretomes derived from MSCs are known to contain many different bioactive substances and growth factors [63]. As a result, they are able to influence and promote several processes, which include not only immunomodulatory and inflammatory responses but also the induction of angiogenesis [64,65,66]. Pre-clinical studies have demonstrated interesting results, but these are difficult to compare due to a lack of standardization [38,67,68,69,70,71,72,73,74], the challenge of the high complexity of blood vessel branching structures [46] and different terminologies used [72] (Table A1). Not only improved concepts are necessary, but also the introduction of an index for the comprehensive assessment of angiogenic activity.

Human adipose tissue-derived mesenchymal stem cells (ADSCs) have the advantage that they can be harvested by a minimally invasive procedure, with little burden to the patient, and have a sufficient proliferation rate [75]. Further investigation of the secretome of these cells is part of numerous experimental studies and clinical trials to explore their therapeutic effects [76,77,78]. For the two examined secretomes in our study, which originate from ADSC-based spheroids, the results could not be more different (Figure 3, Figure 4 and Figure 5). While the group of spheroids large showed a pro-angiogenic effect, the group of spheroids small displayed an anti-angiogenic and therefore an opposite effect. This phenomenon can be attributed to the size of the spheroids used for secretome production. The metabolism of the cells in a spheroid depends, among other things, on the oxygen content. Depending on the size of the spheroid [79,80], cells in the core are exposed to hypoxia because of limited diffusion of nutrients and required gasses [81]. As a result, a necrotic core develops [20,81,82], contributing to increased secretion of bioactive factors [63,83]. Several studies have previously demonstrated that in both monolayer and 3D cultures, hypoxic conditions are directly related to secreted factors and thus to secretome composition [63,81,84]. In addition to an increase in VEGF and FGF2 [22,63,78,81,84,85,86] increased production of ECM components, such as laminin, elastin, collagen I, and fibronectin [20,22,81], has also been demonstrated. Secretion of antiangiogenic factors such as interleukin-4 (IL-4), interferon-gamma induced protein 10 kD (IP-10), platelet factor 4 (PF4), activin A, and dipeptidyl peptidase IV (DPP IV), as well as downregulation of proangiogenic genes, like *IGF1*, matrix metalloproteinase-1 (*MMP-1*), *TGF-β3*, platelet-derived growth factor receptor beta (*PDGFRB*), and placental growth factor (*PGF*), was observed in the context of senescent ADSCs after long-term culture [78]. Rovere et al. discovered increased senescence in spheroids consisting of 2600 MSCs per spheroid after 72 h of cultivation compared to spheroids of 1000 MSCs [87]. The conditioned medium of the larger spheroids contained not only anti- and pro-angiogenic but also pro-inflammatory factors [87]. The extracellular vesicles of the smaller spheroids showed a greater angiogenic potential in vitro [87]. Further investigations are needed to examine the two spheroids of small (250 cells) and large (8000 cells) size in our study and their secretomes in more detail and to analyze these factors, among others. In particular, in our functional study, the secretome of the small spheroids has an inhibitory effect on angiogenesis (Figure 5A,C), representing ideal stimulatory examples for the establishment of a standardized assay with multiple comprehensive readouts.

Another influence on the secretome profile is the mechanical force to which MSCs are exposed during culture [63,88]. The stimulus experienced by the surrounding microenvironment of the cell leads to biochemical responses [63,89]. This fact might be relevant with respect to the angiogenic potential of the three types of secretome tested. The different mechanophysical properties of MSCs in monolayer and 3D cell cultures are due to an altered organization of the cytoskeleton [63,90]. The cell–cell interaction, which is enhanced as a consequence in spheroids, leads to significant differences in gene expression and changes in the composition of the secretome, among other effects [63,90]. The secretomes examined in this study originated from monolayer, spheroid small, and spheroid large cell formats, where it can be assumed that different tensile forces acted on the MSCs in each culture condition [91]. Although the cells in the spheroids small group have had more cell–cell contact than the cells in the single-cell group, no increased proangiogenic potential of the secretome was detected (Figure 3, Figure 4 and Figure 5). This could be due to a lack of hypoxia in the spheroid because of the low cell number of 250 cells per spheroid [92]. In addition, it is likely that the low cell number in gravity-based self-assembly will not result in beneficial mechanical stimulation of the cellular aggregate, leading to a lack of supportive effects for pro-angiogenic therapy. Although several factors that have a significant influence on the secretory profile of cells and thus angiogenic potency are already known [63,93], further fundamental and systematic analyses are needed in this field.

The aortic ring assay is a powerful tool to investigate the angiogenic activity of the secretome, because it is able to detect migration, sprouting, microvessel growth, and lumen formation [3]. These developments begin at the onset of blood vessel formation, even before perfusion is seen in the vessels. In the spheroids large secretome group, branches with a smaller average length were measured compared to the single-cell secretome group (Figure 4 and Figure 5). This indicates that more microvessels were formed with the treatment of spheroids large secretome. These data confirmed results from a previous study, which also showed increased microvessel phenotype in spheroids compared to single-cell group in the CAM assay [23]. The development of sprouts is regulated by notch signaling both in vitro and in vivo [3]. This is confirmed by gene expression of investigated tip cells in vitro, which showed overexpression of *PDGFB* and *VEGFR2*, among others. Endothelial cells downregulate vascular-specific genes over time in favor of upregulation of microvascular genes [3,94]. This could explain the effect in our study that there was only a small increase in length from the initial vessels measured (Figure 3) to the later time points from day 4 to day 7. In contrast, there was hardly any increase in the average branch length during the same period, which can be attributed to the increased formation of microvessels (Figure 4).

**Figure 6:**
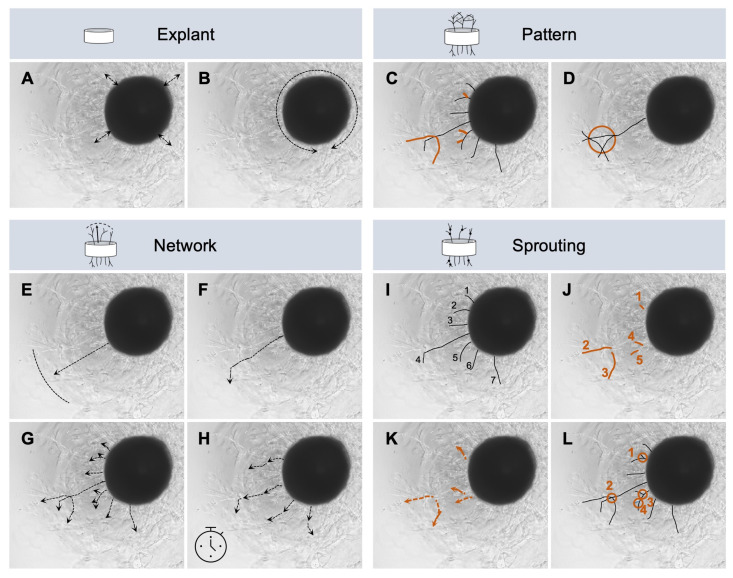
Exemplary visualization of the evaluation of individual parameters. A comprehensive analysis covering the categories explant, pattern, network properties, and sprouting includes the following parameters: migration (**A**) and circumference of aortic ring (**B**), vessel structure (**C**), number of loops (**D**), maximum radial outgrowth (**E**), maximum initial vessel length (**F**), total and mean vessel length (**G**), speed vessel length (**H**), vessel count and vessel density (**I**), number of branches (**J**), mean branch length (**K**), and number of junctions (**L**).

The initial sprouting phase occurs during the first two days of angiogenesis [3]. This is then followed by the formation of a lumen and vessel maturation, forming tight junctions and laying down the basement membrane [3]. In our study, a comprehensive analysis of angiogenic activity was possible from the fourth day of incubation (Figure 3 and Figure 4). When examining samples that exhibit an anti-angiogenic or an inhibitory effect on angiogenesis, it is not possible to investigate earlier time points because of the difference in speed during sprouting. With an initial onset of angiogenesis that is slow on average, this would allow false-negative conclusions to be drawn about the angiogenic potential. Only when equilibrium has been reached and the angiogenic system is established can a reliable statement be made about the potency of the samples under investigation. In our modified AAI [47], the development of the vessels between the fourth and seventh incubation day is therefore included. In addition, it also opens up the possibility of involving different time points according to need using a kinetic, as the detection is a non-invasive imaging method. The individually evaluated time points (Figure 3 and Figure 4) show a scattering of data per time point. This scatter is counteracted when a sample or each aortic ring is evaluated individually. Specimens that show less vascularization on day four may still develop a higher vessel density at a later incubation period compared to their initial situation. Compared to existing vascular or angiogenesis indexes, which were used for the CAM assay but not for the aortic ring assay, these also allow for a comparison over time and normalization over a negative control group [47,48,51]. However, not all indexes are also orientated toward an angiogenesis-inhibiting effect [48]. The values refer exclusively to the number of vascular intersections counted [47,51] or to the number of vessels [48]. The examined secretomes in our study showed without predictive importance factor an increase of 1.089 vessels per mm aortic ring for the vessel density of the secretome of the spheroids large group and 0.630 for the single cells group, while the secretome of the small spheroids showed a decrease in vessel density from day 4 to day 7 of −0.417, respectively (Table S1). Based on an index that is built on one parameter, one of the three secretome groups in our assay showed an anti-angiogenic effect. If the other eleven parameters determined are also taken into account in the evaluation of the secretomes with regard to angiogenic activity, then the secretome of the spheroid large stands out compared to the secretome of the single cells due to long vessels, number of branches, and number of loops (Table S1). Despite vessel density, the secretome of the large spheroids impresses with other outstanding parameters, which show a comprehensive picture of the vessel architecture and exhibit a pro-angiogenic effect in the AAI (Table S1, Figure 5). The number of vessels alone is not the only indication of the development of vascularization. Only long single vessels are not able to provide the same blood flow in a tissue to be regenerated as a complex formed vascular system, consisting of architecture and hierarchy with branches and corresponding microvessels [23,95]. We have, therefore, created an index based on characteristic parameters (Figure 6, Table S1), which allows for a standardized evaluation between the groups. Furthermore, as a novelty in this assay, we have extended the assessment of angiogenic activity by a comprehensive angiogenic profile (Figure 5). This should support the translational approach when it comes to transferring the in vitro data to the in vivo situation, especially since neovascularization is a highly complex orchestrated process.

The harmonization of the methodological evaluation in the form of an index is the first step toward the validation [69], improving the robustness [69,72], and reproducibility [71,73,74] of the results and contributes significantly to the success of the translation of a novel therapy. By obtaining high-quality data, not only the researchers will benefit, but also other stakeholders like sponsors, ethics committees, regulatory agencies, and last but not least the patient [67,72,74]. The clinical impact already starts with the experiments in basic research and with promising preclinical findings [71]. With the AAI and the comprehensive angiogenic profile presented in this paper (Figure 5, Table S1), we introduce a tool to standardize the data obtained using a uniform approach and thereby harmonize prospective assessments of angiogenic studies. Depending on the methodology [37,38] used for the evaluation of the therapeutic agent under investigation, individual tailoring of the subindex parameters will be required. The index currently allows for both, a concise assessment of the angiogenic potential with a few standard measurements, but also the creation of a complex profile based on the selected possible benchmarks. The next step will be to translate the index from ex vivo to in vivo preclinical studies to prove its feasibility and predictability for more complex systems.

The study has some limitations. First, apart from calculating the AAI for each donor (Figure S2), the inter-donor variability was not further examined in detail in this study. Second, the influence of matrigel on the results cannot be excluded. A matrigel-free alternative should be aimed for. Third, the secretomes were not analyzed in detail for their composition. Several studies on MSC-based secretomes and the analysis of their proteome are already available [87,96,97,98,99,100]. After obtaining the anti-angiogenic index for the secretome group of spheroids small, this aspect is also of interest and will be further investigated at a later time. Finally, fourth, a histological examination of the samples was not performed due to a lack of chicken-specific antibodies. However, this would provide additional information on the phenotype of the grown vessels.

In summary, the secretomes produced by the large spheroids demonstrated the formation of microvessels and a complex network structure in the ex vivo aortic ring assay. This secretome was also superior to the secretomes of small spheroids and single cells when it came to their overall angiogenic potential in this assay. The standardized analysis and evaluation of the angiogenic activity allows for the results to be supplemented with an individual angiogenic profile of the sample under investigation to characterize the given vessel network complexity. The three different secretomes proved to be suitable to establish the novel angiogenic profile for the aortic ring assay, as they exhibited angiogenic stimuli that were different enough from each other.

## 4. Materials and Methods

### 4.1. Literature Research

To obtain an overview of possible ways to quantify angiogenesis, a list of parameters used in other pre-clinical studies to evaluate angiogenic assays was assembled as shown in Table A1. For this purpose, the following keywords were searched in PubMed: angiogenic score, angiogenic index, quantification angiogenesis, assessment angiogenesis, aortic ring assay, CAM assay, and tube formation assay. In the second step, the parameters identified in each study were classified into the following five categories: explant, pattern, network properties, sprouting, and cellular level.

### 4.2. Secretome Production

Lipoaspirate was performed (*n* = 3, mean age 39.3 ± 9.7 years) after obtaining the written patient’s consent. The research was carried out according to The Code of Ethics of the World Medical Association (Declaration of Helsinki). The institutional review board has approved the study, and the protocols were conducted in accordance with the Cantonal Ethics Committee Zurich in Switzerland (KEK-ZH-Nr. 2010-0476/0). The isolation and cultivation of the human adipose tissue-derived mesenchymal stem cells (ADSCs) were followed as described elsewhere [101]. For the generation of spheroids, the hanging drop method was used [20]. In the volume of 25 μL complete medium (DMEM high glucose (Sigma-Aldrich, Buchs Switzerland) containing 1% penicillin/streptomycin (Thermo Fisher Scientific/Gibco, Basel, Switzerland) and 10% fetal calf serum (FCS; Thermo Fisher Scientific/Gibco, Basel, Switzerland)), either 250 cells or 8000 cells were cultured to produce two different spheroid sizes (small = 250 cells per spheroid, large = 8000 cells per spheroid). Cells were cultured for three days in Terasaki microtest plates (Greiner bio-one, Frickenhausen, Germany) at 37 °C and 5% CO_2_ until complete spheroid formation occurred. Microscopy and image acquisition were performed using a Zeiss Axio Vert.A1 brightfield microscope with ZEN 2.6 lite software (Carl Zeiss Microscopy, Oberkochen, Germany). The spheroid size was analyzed using ImageJ (version 2.9.0, NIH, Bethesda, MD, USA). For the preparation of the corresponding secretome, spheroids were carefully washed twice with phosphate-buffered saline (PBS; Sigma-Aldrich, Switzerland) in the Terasaki microtest plates and then cultured in 25 μL serum-free medium (SFM) for three additional days. In parallel, hADSC SCs (3000 cells/cm^2^) were also cultured with SFM for three days. Harvested secretome from both spheroid cultures and from SCs was centrifuged (Centrifuge 5702, Eppendorf, Hamburg, Germany) at 2000× *g* rpm and aliquots were frozen at −80 °C. The cultivation of the cells, the production of the small and large spheroids, and the subsequent harvesting of the secretomes were carried out individually for each donor. The cells of the three donors were not pooled at any time. The total protein concentration of the individual secretome groups was determined in triplicate. For this purpose, the secretomes were 10× concentrated with centrifugal filters (Amicon Ultra, Ultracel-3K, Merck Millipore, Cork, Ireland). The DC protein assay Biorad, Hercules, CA, USA) was carried out according to the manufacturer’s instructions. The measurement was performed using a plate reader (Biotek, Agilent, Winooski, VT, USA) at an absorption of 750 nm.

### 4.3. Isolation of Chick Aorta

Until chicken embryonic day 14 no IACUC approval is required according to Swiss animal care guidelines (TSchV, Art. 112). Briefly, fertilized Lohmann white LSL chicken eggs (Animalco AG Geflügelzucht, Staufen, Switzerland) were incubated at 37 °C and 50–70% humidity for 14 days. After 14 days, the eggs were opened carefully, and the chick embryo was taken out and weighed to determine the weight of the embryo (Mettler Toledo, Greifensee, Switzerland). The chest was cut open using surgical scissors and a scalpel. When the heart was uncovered, it was cut from the aorta. The aorta was uncovered, removed, and placed into a Petri dish filled with PBS containing 1% of penicillin/streptomycin solution. Before the aortas were cut in rings of approximately 1 mm thickness under microscopic view and using a ruler, the connective tissue surrounding the aortas was removed carefully, trying not to damage, compress and/or squeeze the aorta. The obtained rings were washed in a Petri dish filled with PBS containing 1% of penicillin/streptomycin and stored at room temperature for later use. Approximately 10–15 rings were obtained from one embryo. Per series (experiments with secretomes from one donor including the control group) five to eight aortas were prepared and the resulting rings were pooled. This procedure does not allow any conclusions to be drawn about the performance of the aortas of an individual embryo and minimizes possible bias due to potential inter-individual variations.

### 4.4. Preparation of the Aortic Ring Assay

Four different matrices, collagen solution from bovine skin (Sigma-Aldrich, Switzerland), jellagel (Jellagen, Cardiff, UK), matrigel (matrigel matrix basement factor reduced; Merck/Corning, Buchs, Switzerland), and chicken egg white were compared. The chicken egg white matrix was produced as described elsewhere [42] by extracting egg white from costumery eggs (Coop Genossenschaft, Basel, Switzerland). Since microparticles were noticed in the egg white disturbing the evaluation purification steps were added. Firstly, the egg white was filtrated through a gauze. Afterward, it was centrifuged for five minutes at 3000 rpm. Several egg whites (*n* = 10) were pooled and then aliquoted and frozen at −20 °C. A 96-well flat bottom plate was prepared with the matrices. For this purpose, 50 μL per well of each matrix was filled on ice to prevent early gelation. Egg white was incubated at 60 °C for 45 min and collagen, jellagel, and matrigel were incubated at 37 °C and 5% CO_2_ for 1 h until a gel-like consistency was achieved. After incubation the prepared aortic rings were placed carefully on top of the solidified matrix. Care was taken to ensure that the lumen was orientated perpendicular to the first layer of the matrix. The tissue was not fixed in the well, and overlaying the next layer of the matrix in the following step may result in a slight change in the position of the ring or lumen, but without influencing the quality of the assay or the results. On top of the aortic rings another 50 μL of matrix solution was added. After a further incubation period, 50 μL per well of secretome, SFM, or complete medium was added to the solidified matrix. The secretome volume was normalized to the lowest cell number at production for all groups and was thus diluted accordingly with SFM. No secretomes were pooled and each secretome per cell format and per donor was tested individually using the aortic ring (*n* = 5 replicates per group and per donor). Afterward, the plates were incubated at 37 °C and 5% CO_2_ and microscopic photo documentation was performed on day 1, day 4, and day 7 (Figure S1). In the first four days, 30 μL of secretome, SFM, or complete medium was added daily to each well.

### 4.5. Evaluation of Angiogenesis

The photo documentation was performed using a Zeiss Axio Vert.A1 brightfield microscope with ZEN 2.6 lite software (Carl Zeiss Microscopy, Germany). In the pictures, different matrices regions of interest (ROIs) were evaluated for comparison. These areas were circular sectors of a circle centered in the aortic ring. In these circular sectors, vessel endpoints were counted manually and the maximum outgrowth radius was measured from the outer edge of the aortic ring with ImageJ (Version 2.9.0, NIH, USA). To extrapolate the counted endpoints for the entire sample, the angle of the circular sector was measured as well. Using the maximum outgrowth radius as well as the angle of the circular sector, a value for the vessel area was calculated.

The comparison of different types of secretome was evaluated by determining a group of different parameters, which were chosen based on the categories explant, pattern, network, and sprouting as listed in Table A1. The following parameters were determined for the category explant (Figure 6A,B): migration of explant and circumference of the aortic ring. Migration of the explant was given when sprouting became visible (Figure 6A). The circumference was determined by measuring the outer circumference of the explant (Figure 6B). The following parameters were determined for the category pattern (Figure 6C,D): vessel structure with a focus on initial vessels and number of loops. The proportion of initial vessels compared to existing branches was determined for vessel structure (Figure 6C, initial vessels shown in black and branches in orange). The loops formed were counted and shown as the number of loops (Figure 6D). If three or more branches were interconnected, forming a circle, this structure was defined as a loop. The following parameters were determined for the category network (Figure 6E–H): maximum radial outgrowth, maximum initial vessel length, total and mean vessel length, speed of mean and maximum vessel length per hour and per day. The value of the maximum radial outgrowth was measured at a right angle from the outer edge of the ring to the tip of the vessel (Figure 6E). This can be a vessel tip from an initial vessel but also from a branch. The highest measured value of all outgrowths was designated as a maximum radial outgrowth. When determining the maximum initial vessel length, the initial vessel length was measured from the outer edge of the ring without taking possible branches into account (Figure 6F). The longest measured initial vessel per ring will represent this parameter. The maximum radial outgrowth and the maximum initial vessel length do not necessarily have to be the same vessels, since depending on the direction of growth of the initial vessels, they can become long but are not aligned straight ahead. To calculate the total vessel length, the sum of all initial vessels and branches was formed and derived from this, and the mean was then calculated based on the number of vessels and branches measured (Figure 6G). Based on the growth of the vessels from day 4 to day 7 and the previously determined values of the mean and total vessel length, the mean and maximum vessel length per hour and per day were calculated using the exact time that elapsed between taking the microscopic images on days 4 and 7 (Figure 6H). The following parameters were determined for the category sprouting (Figure 6I–L): vessel count and vessel density, number of branches, mean branch length, number of junctions, and junctions per vessel. For the vessel count, the number of initial vessels determined without branches was taken into account (Figure 6I). The number of individual branches was shown as the number of branches (Figure 6J) and the mean branch length was calculated based on the length of the branches (Figure 6K). Junctions were defined if at least one branch originated from an initial vessel and the number of junctions was shown as a number of junctions and as junctions per vessel (Figure 6L). For the latter value, the number of junctions was related to the vessel count. All parameters (Figure 6) were measured manually using the software ImageJ (version 2.9.0). For each donor, each secretome of the corresponding cell format, and each time point, the values were first determined individually and then recalculated with all donors as a group in order to obtain an indication of the complexity of the vessel network per secretome group without being able to draw conclusions about individual donors (Table S1).

Based on the values determined on days 4 and 7 the angiogenic profile was established, and the AAI was calculated using the following Equation (1), which was adapted from Demir et al. [47]:

(1) $$\mathrm{AAI}=\frac{\left\{{AR}^{7thDoT}\left[Treatment\right]-{AR}^{4thDoT}\left[Treatment\right]\right\}-\left\{{AR}^{7thDoT}\left[Control\right]-{AR}^{4thDoT}\left[Control\right]\right\}}{\left\{{AR}^{7thDoT}\left[Control\right]-{AR}^{4thDoT}\left[Control\right]\right\}}$$

where *AR* represents the angiogenic response of the respective examined parameter, and *DoT* represents the day of treatment with the secretome group (Treatment) or serum-free medium group (Control). The original DAAI [47] was modified for this study with regard to the treatment days specified, the terminology amended, and the parameters to be determined. For the calculation of the subindices (Equation (2)), AAI for each parameter (Table S1) were obtained separately, weighted, and summarized as follows:

(2) $$\mathrm{AAI}\;\mathrm{for}\;\mathrm{subindex}=\frac{1}{n}\sum_{i=1}^{n}{AAI}_{i}$$

where *n* is the number of parameters and *AAI_i_* refers to the individual weighted values of AAI. The values of the predictive importance factors were based on the potential meaningfulness for the evaluation of angiogenesis, in which a predominantly complex vascular architecture corresponds to the natural occurrence. The final *AAI* was calculated based on the mean of the subindices (Equation (3)):

(3) $$\mathrm{Final}\mathrm{AAI}=\frac{1}{n}\sum_{i=1}^{n}{SI}_{i}$$

where *n* is the number of subindices and *SI_i_* represents the individual subindex values.

### 4.6. Statistical Evaluation

The data in the text are represented as means ± standard deviations (SD). The data were analyzed and visualized with Microsoft Excel (version 16.77) and GraphPad Prism software (version 10.0.2). Comparisons between groups were performed with one-way ANOVA with Tukey’s post hoc test or Kruskal–Wallis test followed by Dunn’s multiple comparison test whenever appropriate. A *p*-value < 0.05 was considered statistically significant. The Shapiro–Wilk test was performed to analyze normality, and the ROUT test was employed to identify outliers.

## 5. Conclusions

A comprehensive characterization of the vessel network after ex vivo incubation with three secretome groups single cells, spheroid small and spheroid large was performed using AAI and angiogenic profile. While the secretome of the spheroid small group showed an inhibitory effect on angiogenesis, the spheroid large group was convinced with a complete pro-angiogenic profile, and a higher AAI compared to the single cells group. The secretome of large ADSC-based spheroids offers potential for therapeutic applications in regenerative medicine.

## Data Availability

Datasets acquired and analyzed during the current study are available from the corresponding authors on reasonable request.

## Supplementary Materials

The following supporting information can be downloaded at: https://www.mdpi.com/article/10.3390/ijms26010291/s1.

## Appendix A

**Table A1:** Characterization of neovascular phenotype in ex vivo culture based on categories. The column synonyms refer to terms that were used synonymously with the respective parameters in the cited studies.

Category	Parameter	Synonyms	Definition	Evaluation	Benefits	Disadvantages	References
Explant	Outgrowth	n/a	Migration of progenitor cells and response to culture condition	Number of explants with a neovascular outgrowth over total explants	Quick overview of the quality of explants and matrices, an early indicator for technical problems throughout the experiment	The significance of the parameter decreases with a small number of samples	[102,103]
	Area	n/a	Area (*A*) of the projection of the aortic ring into the image	*A* in mm^2^ equals the number of pixels that form the object multiplied by a calibration constant square	*A* has almost the same value for each set of experiments and may serve for standardization	Software (e.g., ImageJ) for the evaluation and for the generation of binary images is necessary, *A* depends on the age and size of the animal	[44]
	Circumference	Size	Length of the line that delimits the vessel	Measurement of the outer perimeter of the vessel from captured images	The vessels develop exclusively at the cutting edge of the explant, explant circumference can be used for normalization	Depends on the age and size of the animal	[104,105]
	Shape	Form	Shape factor (*F*) of arterial ring	Perimeter^2^/4π*A* describes the deviation of an object from a true circle (*F*), it gives a minimal value of 1 for a circle and larger values for shapes having a higher ratio of perimeter to area	*F* has almost the same value for each set of experiments, results do not depend on the geometry of aortic rings	Software (e.g., ImageJ) for the evaluation and for the generation of binary images is necessary	[44,105]
Pattern	Mesh	Cellular organization, morphology	Quantification of cellular organization, which are closed areas delineated by segments and associated junctions	Use of extension of “Angiogenesis Analyzer” plugin for ImageJ software, results for mean size of meshes and total mesh area	Suitable as an early marker, as over time, segment interruptions resulting in mesh fusions increase	Software-based evaluation of the vectorial objects through corresponding algorithms, formed network often varies in different physiological and pathophysiological environments	[16,95,106]
	Structure	Tree detection, branching pattern, microvessel distribution	Geometry of microvessels as a function of distance to the aortic ring, or as a skeletal illustration	Tree modeling consists of segmentation followed by skeletonization of vascular structures, number of intersections of microvessels with a grid defined as the successive boundaries of the dilated aortic ring	Both qualitative (skeletonization) and quantitative (microvessel distribution) evaluation is possible	Manual evaluation is not feasible, qualitative evaluation is based on a subjective comparison of the images by eye and shows junctions without counting the branches, avoid shadows due to phase contrast lighting and small acellular structures during segmentation	[16,44,46]
	Angle	Degree of branching vessel	Angle between branches	Measurement of value in degrees of how two branches relate to each other	Provides information about vascular patterns, network formation, and parallel alignment of vessels	Software-based (e.g., Synedra View) evaluation is needed	[95]
	Loop	Endothelial network loop, loop formation, vessel lumen	Loops with at least three sides	Count of endothelial network loops	Quantification identifies effects on the development of endothelial networks	A cell counter tool is recommended, inadequate or uneven polymerization of the matrix, as well as its damage, can interfere with successful network development	[8,106,107]
Network properties	Vessel area	Sprouting area, network area, vascular area outgrowth area, tubule area	Total area quantifies the surface of all measured vessels	Based on the measured length and width of vessels, the area can be calculated, the value can be given as a total value per area or region or as a percentage, as well as a relative value, whereby the vessel area at time T = 0 is subtracted from the vessel area after further corresponding treatment days	Vessel area increases as the vascular network grows, the relative vessel area enables comparison over time	Use of image processing software (e.g., IKOSA, Wimasis) as the measurement of the surface area of all vessels cannot be determined by manual analysis, new vascular sprouts formed during angiogenesis are thin and may not affect the total vessel area as much as other parameters	[1,8,46,106,108,109,110,111,112]
	Radial outgrowth	Radial network growth, circumference of the arterial ring, aortic ring area, sprouting area, maximal distance migrated, area of migrated vessels, microvessel outgrowth	Distance from the aortic ring to the furthest distal outgrowth of cells	Measuring the distance from the cut end of the aortic segment to the approximate mean point of vessel growth	Value correlates with the apparent number of cells forming vessels, same images can be used to quantify network properties, including radial outgrowth and loop formation in one assay quadrant	Dependent on the growing pattern, less informative value than the total vessel length	[8,44,103,110,113,114,115,116,117,118]
	Vessel length	Sprout length, tubule length, microvessel length, length of capillary-like microtube, segment length, branch length	Length of vessel centerline	Determination of the length of individual vessels, including their branches, in an image using a software application, values can be expressed as the sum of all lengths (total vessel length), the average vessel length, the average length of segments, which includes the distance between junctions, and the maximum vessel length	Vessel elongation is a critical process during angiogenesis, the total length of the vascular network is influenced by the appearance of new branches and the elongation of existing vessels, widely used parameter with a high informative value, vessel length is an important parameter for measuring the effects of exogenous factors on angiogenesis, length of vascular tree is associated with a later stage of development	Difficult to analyze with high vessel density and overlapping of vessels, many studies focus on assessing the larger vessels while paying little attention to microvessels, vessel length can be affected by factors such as cell adhesion or chemoattractant gradients	[1,5,8,11,16,44,46,95,103,104,105,106,108,110,111,112,115,118,119,120,121,122,123,124]
	Vessel thickness	Vessel diameter, width vessel, wall thickening	Diameter of a vessel	Determine thickness by measuring the diameter of a vessel	Changes in the mean thickness of the vasculature are related to angiogenic processes, vessel width reveals primary and secondary orders, leading to the identification of main and minor vessels of the vascular network	Decrease in the mean diameter of the vascular network through the development of new thin sprouts	[1,46,95,108,109]
	Speed	n/a	The ratio of distance covered to time spent	Measurement over time of prominent vessel elongation in living culture	Examination of vascular proliferation possible	Not suitable for a low degree of vascular development	[107]
Sprouting	Vessel counts	Vessel density, number of microvessels, tube number, number of capillaries	Cells are organized as vessels after endothelial-driven cells sprouting from the aortic ring, and the number of vessels is counted accordingly, total vessel number based on branch and terminal points counting	Documentation by phase contrast microscopy followed by manual or computer-assisted counting of vessels (per field of view or per ring), values can be displayed as the total number of vessels (vessel counts), vessel density (total number of vessels per area), or the number of vessels per aortic ring	Widely used parameter with a high informative value, number of vessels is an important parameter for measuring the effects of exogenous factors on angiogenesis, the potency of angiogenic activity can be assessed by the number and growth rate of newly formed vessels and calculated as an angiogenic score (vessel density in relation to distance from the ring)	Difficult and time-consuming when counted manually, comparability between studies may be difficult due to different ways of presenting the values, accurate quantification based on visual counts may not be possible, as the number of neovessels and the complexity of the vascular networks in the culture may be very high	[1,5,11,44,95,102,103,105,106,107,108,110,118,119,120,122,123,124,125]
	Branch	Sprout, segment	Line that is either connected to the main vessel by a junction or is located between two junctions	Counting the number of branching in vessels (manually or computer-assisted)	New branches develop by sprouting from pre-existing vessels, the number of branches provides information on the way the vessels are organized and develop in the presence of stimulators or inhibitors of angiogenesis	No minimum inclusion criteria exist on how many aligned cells a branch must consist of (e.g., three or more cells), no distinction is made in the manual evaluation as to whether extremities or connections within the vascular tree are involved	[1,11,44,46,95,105,106,108,113,118,120,123]
	Junction	Branching point, node, intersection	Junction consists of a minimal structure and allows branching in a skeletonized network, junction is determined where it has at least three adjacent branches	Counting the number of junctions, the values can be presented as the total number of junctions per ring or per field of view, as the average number of junctions per vessel, or as the ratio average number of junctions per average number of branches	It allows a statement on the degree of branching and thus on the architecture of the vessel tree, manual as well as computer-assisted evaluation is possible	In case of overlapping of vessels, an evaluation can no longer be performed accurately, and significance loses its validity, manual count of junctions is very time-intensive	[8,16,106,123]
	Break-away capillaries	n/a	The foremost segments of the sprouts can detach from the parent microvessels and migrate as isolated break-away capillaries on the advancing front of the outgrowth	Quantitation of break-away capillaries based on light microscopic images	This effect is particularly evident in a mid- or long-term culture, a microvessel culture with a small number of break-away capillaries shows thicker and more mature capillaries	No intensively studied parameter in the in vitro setting so far, data available for collagen gel culture of rat aorta	[119]
Cellular level	Endothelial cells	ECs	Flattened cell type that forms a layer covering blood vessels	Staining of cells without or with aortic ring (e.g., GFP, Dil-Ac-LDL) prior to the experiment for in vitro cell tracking possible or afterward for identification of this specific cell type (e.g., CD31, vWF, BSL-B4, BSL-1), analysis by fluorescence microscopy (including capturing Z-series image stacks), calculating the number of ECs aligned in vessels, an area with the maximal sprouting, or number of branches	Additional evaluation of further parameters that characterize the structure of the vascular network, e.g., β-catenin interacts in ECs, and its loss may reduce junction stability followed by increased vascular permeability, closer examination of tip and trunk cells possible, the aortic ring sprouts anatomically mimic microvessels in vivo, making them suitable for studies of paracrine signaling between endothelial cells, pericytes, and smooth muscle cells	Fibroblasts may first have to be removed by a short digestion of the sample, after transfection, the expression of the exogenous gene often decreases at the time when its presence could be relevant for the morphological changes in the cells, after transduction and depending on the titres used, cells may show an altered morphological pattern of sprouting and vascular formation, in contrast to retroviruses, adenoviruses stress the cell through the transcription and translation of adenoviral genes, the range of antibodies available depends on the species to be investigated	[3,44,110,113,116,124,126,127,128]
	Fibroblast-like cells	Pericytes, smooth muscle cells, myofibroblasts	Capillary-like structures with isolated fibroblast-like cells, primarily confined in peri- aortic location, spatially isolated contractile cells on capillaries, play an important role in the main- tenance of vascular and tissue homeostasis	Specific staining enables the determination of the cell type through the exclusion criterion, selectively uptake of fluorescent Dil-Ac-LDL by endothelial cells, remaining fibroblast-like cells are detected as unstained, counting the number of pericytes covering a 100-um-long stretch of neovessel	Myofibroblast-endothelial interactions can be modeled (transduction of EC with a mCherry retroviral vector, and myofibroblasts with a GFP retroviral vector), GFP-labeled pericytes can be utilized to track motility and the recruitment of pericytes into the EC tubes can be observed explant assays are considered to come closest to mimicking the in vivo situation because they include the surrounding nonendothelial cells (such as smooth muscle cells and pericytes) and a supporting matrix, the EC tubes in EC pericyte co-cultures become much narrower and more elongated compared to EC-only tubes, which become wider and less long over time	Pericyte antibody panel is not available for every species	[3,8,37,44,129,130,131,132]

Key: *A*: Area; BSL-1: *Griffonia (Bandeiraea) Simplicifolia* Lectin I; BSL-4: *Griffonia Simplicifolia* Lectin I (GSL I) Isolectin B4; Dil-Ac-LDL: 1,10-Dioctadecyl-3,3,30,30-tetramethyl-indocarbocyanine perchlorate; EC: Endothelial cell; *F*: Factor shape; GFP: Green fluorescent protein; n/a: Not appropriate; vWF: Von Willebrand Factor.
