# Supplementary material for: Standardization to Characterize the Complexity of Vessel Network Using the Aortic Ring Model
Source: Int J Mol Sci. 2024 Dec 31;26(1):291. doi: 10.3390/ijms26010291 (PMC11719671; doi:10.3390/ijms26010291)
Supplement: Supplementary file 1 [file ijms-26-00291-s001.zip › ijms-3340625-supplementary.pdf]

Supplementary Materials

Standardization to characterize the complexity of vessel network using the aortic ring model

Petra Wolint[1,2#*], Silvan Hofmann[2#], Julia von Atzigen[2], Roland Böni[3], Iris Miescher[2], Pietro Giovanoli[2], Maurizio Calcagni[2], Maximilian Y. Emmert[4,5,6,7] and Johanna Buschmann[1,2*]

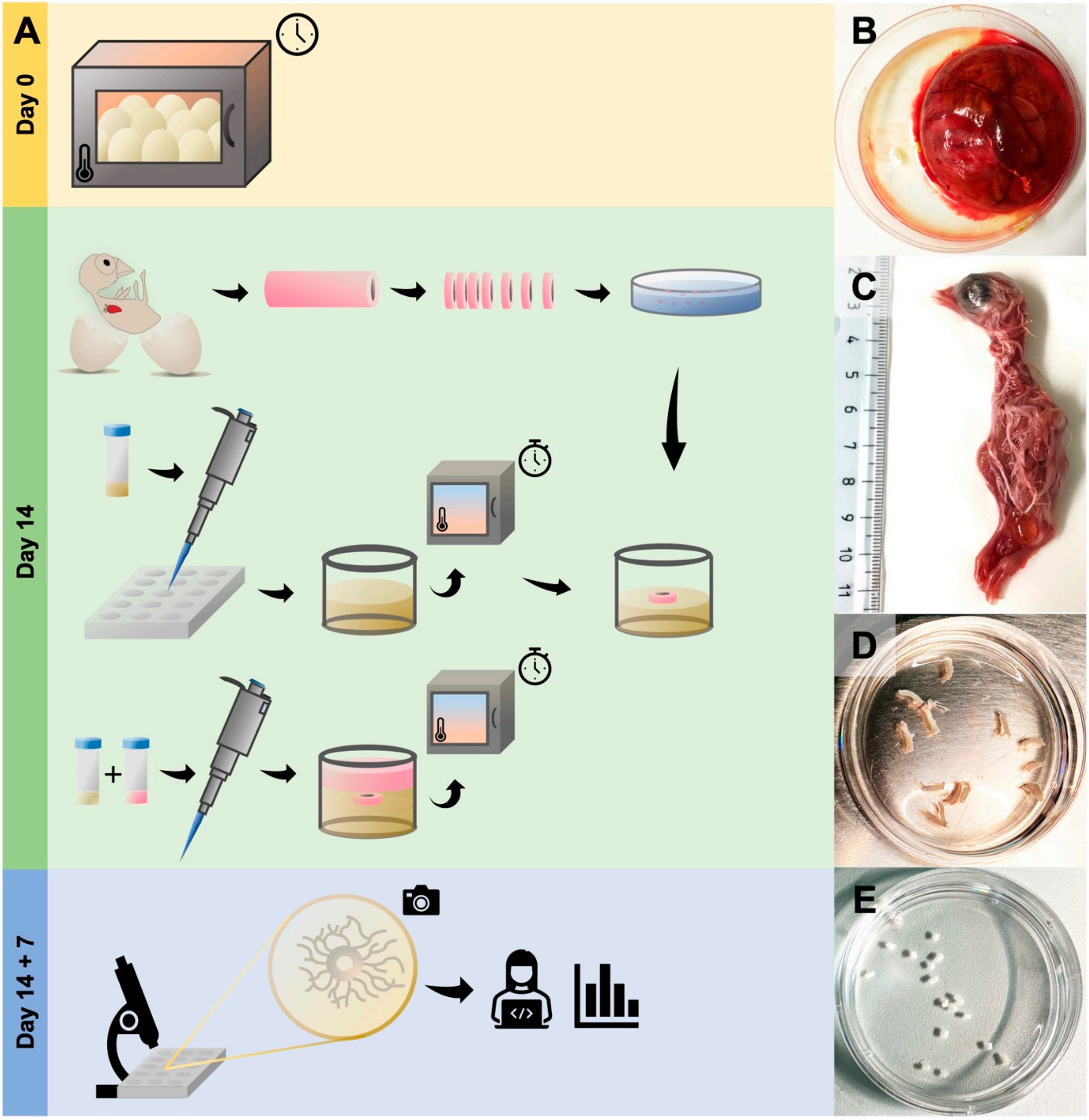

Figure S1. Schematic experimental setup for the assessment of angiogenic potential of secretomes using the chicken aortic ring assay. Schematic overview (A) shows the incubation of fertilized chicken eggs for 14 days. During these days the eggs were turned around twice daily. After 14 days the eggs were opened carefully (B), and the chick embryo (C) was taken out. The chest of the embryo was cut open with surgical scissors and the aorta was removed (D). Afterwards the aorta was cut in rings of approximately 1 mm thickness (E). The rings were placed in a petri dish filled with phosphate buffered saline. A 96-well plate was prepared with the matrix solutions. For this purpose, 50 ml of the matrix solution was placed in a well using a pipette followed by an incubation time of 45 - 60 min (depending on the respective matrix). After the incubation the prepared aortic rings were placed carefully in the wells with the solidified matrix solution. On top of the aortic rings another 50 ml of matrix solution was pipetted and with repetition of the incubation step. Finally, 50 ml per well of secretome or serum-free medium as a control were added. Afterwards the plates were incubated at 37 °C for 7 days and

microscopic pictures were taken on a regular basis. The samples to be tested were added daily for the first four days. The pictures were used to perform the evaluation of angiogenesis and to calculate the angiogenic activity index (AAI) and establish the angiogenic profile.

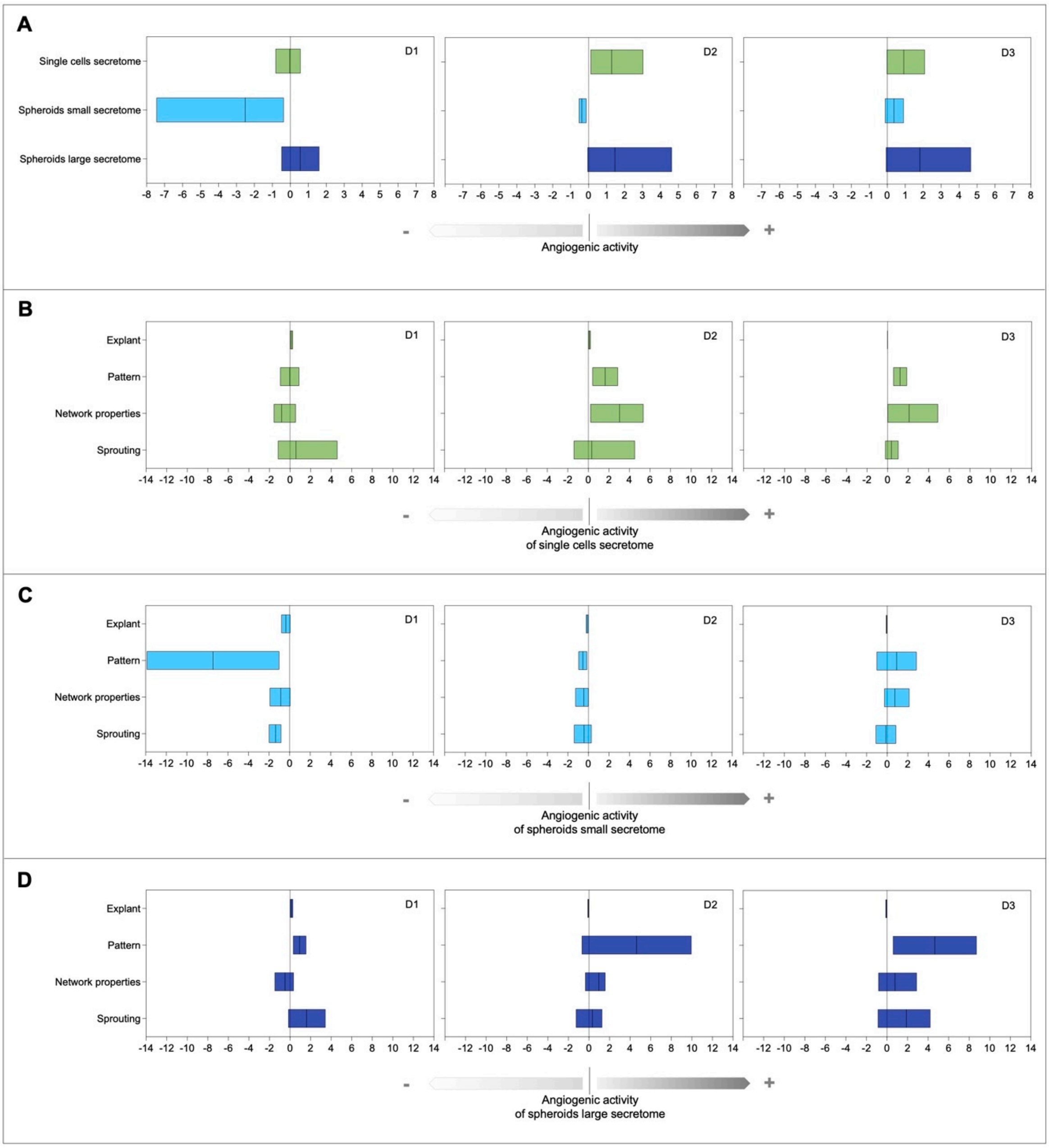

Figure S2. Overview of the angiogenic activity index (AAI) of three secretome groups. The AAI (A) is determined from the values of the sub-index categories explant, pattern, network properties, and sprouting (B-D). Secretomes based on the cultivation of human adipose tissue-derived stem cells (hADSCs) either as single cells (B), as spheroids small (C) or as spheroids large (D) were examined for their angiogenic activity using the aortic ring assay. The secretomes of the three hADSCs donors D1, D2 and D3 were analyzed for each cell format. Data shown as boxplots with min to max values and mean. The value zero represents the angiogenic activity of the control group, with a value of 1 representing an increase of 100 %. The scale range with negative values shows a decrease or inhibition of angiogenic activity.

Table S1. Angiogenic activity index (AAI) was calculated for the different parameters. The values refer to the development of angiogenesis from day 4 to day 7 and in relation to the control group. The weighted values were used for the final AAI.

		Subindex score (unweighted)				Subindex Score (weighted)		
**Subindex**	**Parameter**	**Single cells secretome**	**Spheroids small secretome**	**Spheroids large secretome**	**Predictive importance factor**	**Single cells secretome**	**Spheroids small secretome**	**Spheroids large secretome**
Explant		0.077	-0.111	0.008		0.077	-0.177	0.008
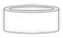	Migration	0.000	-0.133	0.000	2	0.000	-0.267	0.000
	Circumference	0.155	-0.088	0.015	1	0.155	-0.088	0.015
Pattern		0.681	-1.032	3.288		0.948	-2.353	3.410
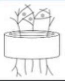	Vessel structure	0.534	-2.642	0.244	2	1.068	-5.284	0.488
	Number of loops	0.827	0.579	6.332	1	0.827	0.579	6.332
Network properties		1.230	-0.075	0.398		1.475	-0.156	0.544
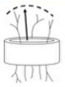	Maximum radial outgrowth	0.670	-0.294	-0.173	1	0.670	-0.294	-0.173
	Maximum initial vessel length	1.615	-0.002	0.532	1	1.615	-0.002	0.532
	Total vessel length	0.025	-0.526	0.575	2	0.051	-1.051	1.151
	Mean vessel length	1.502	0.193	0.328	1	1.502	0.193	0.328
	Speed maximum initial vessel length	2.118	0.136	0.830	1	2.118	0.136	0.830
	Speed mean vessel length	1.448	0.040	0.298	2	2.896	0.079	0.595
Sprouting		0.534	-0.451	0.664		0.427	-0.679	1.285
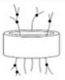	Vessel density per mm aortic ring	0.056	0.031	1.493	2	0.113	0.062	2.987
	Number of branches	-0.298	-0.619	0.935	2	-0.596	-1.238	1.870
	Mean branch length	2.974	-0.747	0.036	1	2.974	-0.747	0.036
	Number of junctions	-0.289	-0.555	0.680	2	-0.577	-1.111	1.359
	Number of junctions per initial vessels	0.224	-0.362	0.176	1	0.224	-0.362	0.176
**Final Angiogenic Activity Index (AAI)**						**0.732**	**-0.841**	**1.312**
